# X-ray directional dark-field imaging using *Unified Modulated Pattern Analysis*

**DOI:** 10.1371/journal.pone.0273315

**Published:** 2022-08-29

**Authors:** Ronan Smith, Fabio De Marco, Ludovic Broche, Marie-Christine Zdora, Nicholas W. Phillips, Richard Boardman, Pierre Thibault

**Affiliations:** 1 Faculty of Engineering and Physics Sciences, University of Southampton, Southampton, United Kingdom; 2 Department of Physics, University of Trieste, Trieste, Italy; 3 Elettra Sincrotrone, Trieste, Italy; 4 European Synchrotron Radiation Facility, Grenoble, France; 5 Paul Scherrer Institut, Villigen, Switzerland; 6 Department for Electrical Engineering and Information Technology, ETH Zürich, Zürich, Switzerland; Rutgers University Newark, UNITED STATES

## Abstract

X-ray directional dark-field imaging is a recent technique that can reveal a sample’s small-scale structural properties which are otherwise invisible in a conventional imaging system. In particular, directional dark-field can detect and quantify the orientation of anisotropic structures. Here, we present an algorithm that allows for the extraction of a directional dark-field signal from X-ray speckle-based imaging data. The experimental setup is simple, as it requires only the addition of a diffuser to a full-field microscope setup. Sandpaper is an appropriate diffuser material in the hard x-ray regime. We propose an approach to extract the mean scattering width, directionality, and orientation from the recorded speckle images acquired with the technique. We demonstrate that our method can detect and quantify the orientation of fibres inside a carbon fibre reinforced polymer (CFRP) sample within one degree of accuracy and show how the accuracy depends on the number of included measurements. We show that the reconstruction parameters can be tuned to increase or decrease accuracy at the expense of spatial resolution.

## Introduction

Dark-field X-ray imaging maps the ultra-small angle scattering caused by inhomogeneities in the local electron density inside a sample [1]. The dark-field X-ray signal originates from features which are smaller than the spatial resolution of the imaging system and are therefore undetectable with conventional absorption and phase-contrast imaging modalities [2]. The growing list of applications includes fields within medical and materials sciences, for example the detection of impact damage [3] and manufacturing defects [4] in composite materials, monosodium urate crystals in murine gout [5], and early diagnosis of lung emphysema in chronic obstructive pulmonary disease (COPD) [6, 7]. Recently, dark-field X-ray has even been demonstrated to be compatible with existing medical CT technology [8].

Commonly, the X-ray scattering profile varies across the sample and is hence anisotropic. This anisotropy is observed in small-angle x-ray scattering (SAXS) measurements and can be used, for instance, to determine the orientation of collagen bundles [9] or of fibres in a composite material [10]. Scanning SAXS can create quantitative images of the scattering anisotropy [11, 12], which can further be combined with tomographic techniques to create 3D vector or tensor fields [13, 14]. Since scanning SAXS imposes stringent requirements on the beam size as well as requiring the sample to be raster-scanned [15], the development of new directional dark-field (DDF) methods is an active research topic. Simplicity, reliability and speed are some of the main parameters being optimised by the imaging community, along with work on translating the technique from synchrotron to laboratory sources. Probably the most established technique is grating-based imaging [1, 16, 17], which is naturally sensitive to the scattering component perpendicular to the grating structures. Circular gratings have also been manufactured for DDF imaging [18, 19]. Beam tracking [20] is also capable of measuring the directional dark-field signal.

Here we present a new approach for DDF imaging based on speckle-based X-ray imaging (SBI). SBI is an experimentally simple to implement, robust technique, initially developed for metrology and phase-contrast imaging [21, 22]. The method relies on the detection of the small sample-induced distortions in a randomly patterned illumination. This pattern is normally produced by a diffuser inserted into the beam. Refraction of the incident radiation by the diffuser particles and subsequent self-interference of the beam produces near-field X-ray speckle [23]. The X-ray attenuation caused by the sample appears as a change in pattern intensity, while refraction results in a lateral shift of the speckles. The dark-field signal manifests itself as a local reduction in speckle visibility caused by ultra-small angle scattering [24].

X-ray speckle-based DDF imaging was first implemented by Wang et al. in 2015 [25]. However, their approach, which is based on scanning the diffuser in small equidistant steps, requires a high degree of setup stability as well as high-accuracy and high-precision scanning stages. Furthermore, hundreds of scanning points are needed, which leads to extremely long scan times. In 2018, Zhou et al. [26] demonstrated speckle-based DDF imaging using a speckle-tracking technique whereby only a single pair of images of the speckle pattern with and without sample were compared, greatly reducing the scan time and requirements on the setup. More recently, Pavlov et al. [27] introduced a DDF SBI technique based on the Fokker-Planck equation using speckle-tracking. This technique extracts the signal directly by modelling the X-ray energy flow within the sample. Wang et al. [28] have introduced another technique which they call Omnidirectional Dark-Field Imaging, where the diffuser is scanned in a spiral pattern. This also gives insight into directional scattering in a sample, however the directional dark-field is measured by analysing the speckle pattern along a discrete number of angles, which may limit the angular sensitivity of the technique. For a more in-depth comparison of speckle-tracking and speckle-scanning techniques (with a focus on phase imaging), we refer to Ref. [29].

In this paper, we present a new method based on the Unified Modulation Pattern Analysis (UMPA) technique [30], which combines information from an arbitrary number of diffuser positions to allow for the extraction of DDF images. We refer to the modified algorithm as UMPA-DDF.

## Materials and methods

**Fig 1:**
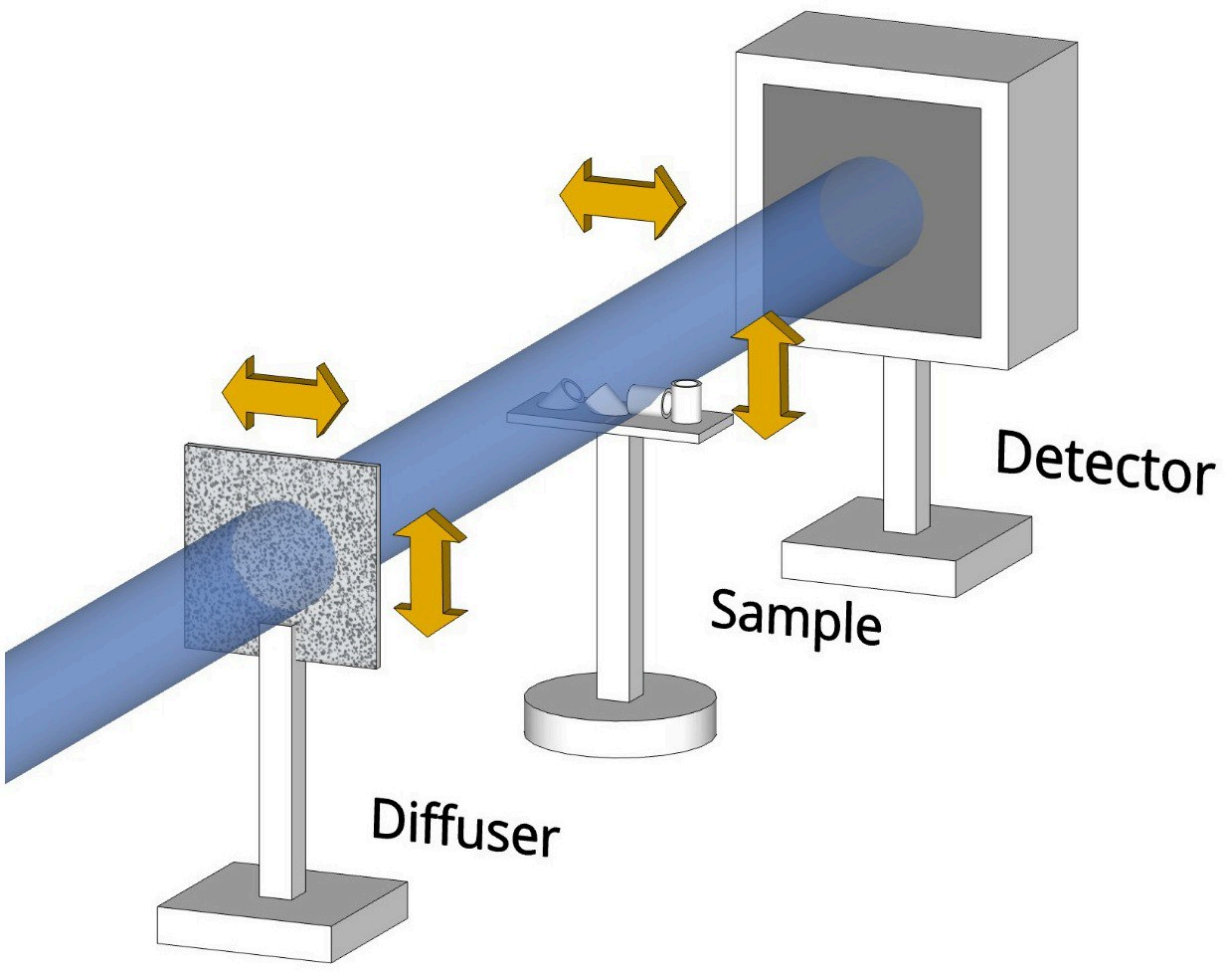
Schematic of a typical experimental setup used for SBI. The X-ray beam is patterned by the diffuser mounted on a scanning stage upstream of the sample stage. A detector system consisting of a pixel array detector scintillator-coupled to an optical microscope, giving sufficient resolving power to resolve the speckle pattern, is used to collect the images.

The setup required for SBI using the UMPA approach, shown in Fig 1, is identical to a standard full-field microscope setup, with the addition of a translation stage, placed upstream of the sample, to move the diffuser. An image of the diffuser only, called the reference frame, is acquired. The sample is then moved into the beam, and a sample image is taken. A full dataset is built by the acquisition of one or more such sample/reference image pairs, taken at different diffuser positions.

**Fig 2:**
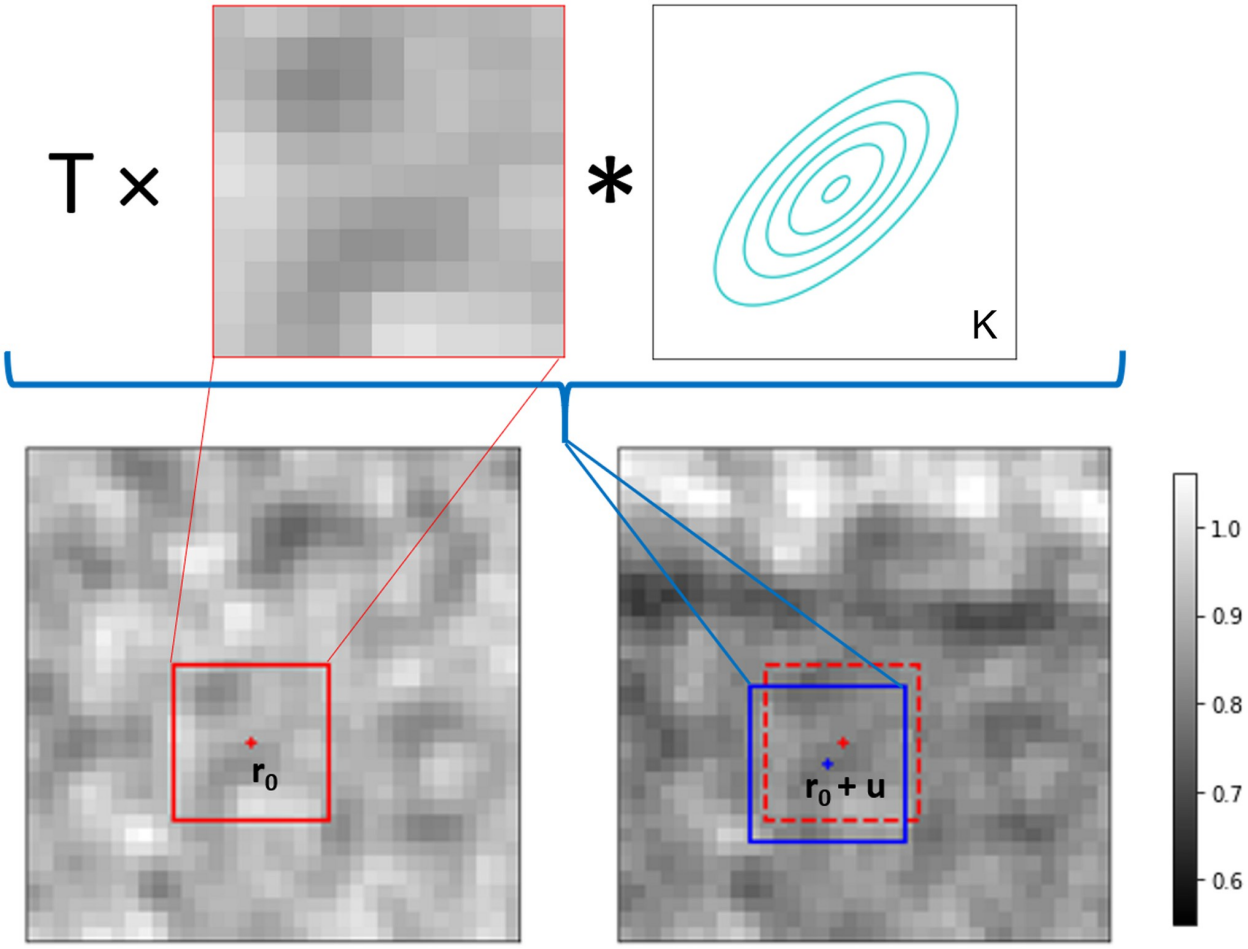
Graphical representation of the directional dark-field UMPA model: The speckle pattern is locally modulated by the sample transmittance (*T*), a convolution with a blurring kernel (*K*_α_) and a lateral shift (u). The transmittance corresponds to an image similar to the one that would have been acquired without the sandpaper in the beam. The lateral shift is used to generate differential phase-contrast images, while the parameters of *K*_***α***_ quantify the dark-field signal. (The blurring kernel and vector shifts shown have been exaggerated).

Fig 2 visualises the image formation model. The sample transmission *T* at pixel **r**_0_ is modelled as a drop in the intensity, while refraction is modelled as the local displacement **u** of the speckle pattern, which is identical to the UMPA model used in our previous work [30]. In this original UMPA formulation, the dark-field signal is modelled as a local reduction of speckle visibility. Here, we use a more realistic model for sample scattering, which is approximated with a convolution of the reference pattern with a two-dimensional blurring kernel. We model this kernel with an anisotropic Gaussian function, with parameters ***α*** = (*α*_1_, *α*_2_, *α*_3_):

$$\begin{array}{c} K_{\alpha }\left(x,y\right)=A\text{exp}[-\frac{1}{2}(\alpha_{1}x^{2}+2\alpha_{2}xy+\alpha_{3}y^{2})], \end{array}$$ (1)

where *A* is a normalisation constant.

Analogous to the original UMPA implementation, the estimation of the parameters (*T*, **u**, ***α***) at a given pixel position **r**_0_ is extracted from the measured data with the minimisation of a least-squares type cost function localised through a window function *Γ*(**r**):

$$\begin{array}{c} L\left(r_{0};T,u,\alpha \right)=\sum_{r}\sum_{j=1}^{N}\Gamma \left(r-r_{0}\right){\left|I_{j}\left(r\right)-T\cdot \left[I_{0,j}✱K_{\alpha }\right]\left(r+u\right)\right|}^{2}. \end{array}$$ (2)

The square term in this sum is the residual, given by the difference between the sample frame intensity *I*_*j*_ and the reference frame *I*_0,*j*_ distorted according to the model at diffuser step *j*. The expression is summed over all *N* frame pairs at the different diffuser positions. (Note that although Fig 2 shows a uniform window function *Γ*, in reality a Hamming window function is used).

As with the original UMPA implementation, the optimisation gives the transmission map *T*, and the differential phase contrast ∇*ϕ* ∝ **u**. The additional three optimisation parameters ***α*** can be used to extract the kernel width of the major axis *σ*_ m_, and minor axis *σ*_ m_, along with the angle *θ* between the major axis and the *x* axis. The transformation from one system to the other is given by:

$$\begin{array}{c} \theta =\frac{1}{2}\text{arctan}(\frac{2\alpha_{2}}{\alpha_{1}-\alpha_{3}}), \end{array}$$ (3)

$$\begin{array}{c} \sigma_{M}^{2}=\frac{1}{2(\alpha_{1}\;{\text{sin}\;}^{2}\theta +2\alpha_{2}\;\text{sin}\;\theta \;\text{cos}\;\theta +\alpha_{3}{\text{cos}}^{2}\theta )}, \end{array}$$ (4)

$$\begin{array}{c} \sigma_{m}^{2}=\frac{1}{2(\alpha_{1}\;{\text{sin}}^{2}\;\theta -2\alpha_{2}\text{sin}\theta \;\text{cos}\;\theta +\alpha_{3}{\text{cos}}^{2}\theta )}. \end{array}$$ (5)

We have observed that convergence of the minimisation of Eq 2 can be difficult to reach in the space of parameters spanned by (*α*_1_, *α*_2_, *α*_3_). This behaviour might be caused by the exponentially decreasing sensitivity of the convolution term with the increase of *α*_1_ and *α*_3_. We obtain the best convergence by using a multi-resolution approach, i.e. through an optimisation that begins at a coarser scale and successively cascades to finer scale. Code for implementing this can be found in S1 and S2 Files. The code takes advantage of a rewritten version of UMPA in C++, providing a vast improvement in speed compared to the original implementation. The processing time for the data described in the following section was 3 hours and 11 minutes on a desktop computer (AMD Ryzen Threadripper 1950X 16-core CPU) with a window size of 7 × 7 pixels, 25 diffuser positions and an image size of 1960 × 560 pixels. (Increasing the number of frames or analysis window size increases the processing time.) This compares to 26 minutes for a 961 × 961 pixel image and 50 diffuser positions for Wang et al. on a similar desktop PC [31].

## Results and discussion

The UMPA-DDF approach was experimentally verified with data taken at the ID19 beamline of the European Synchrotron Radiation Facility (data available in S3 File). A sample was prepared from four pieces of hollow pultruded unidirectional CFRP tube with a 10 mm outer diameter and 8 mm inner diameter (purchased from Easy Composites, UK). These pieces were mounted on a wooden support and arranged so that the fibres ran approximately vertically, horizontally, and along both diagonals.

CFRP, which is composed of carbon fibres between 5 and 7 μm in diameter bound together by a matrix [32], is known to give an anisotropic dark field signal. The pultrusion process used to manufacture these tubes consists of passing the fibres through a heated die while under tension [33, 34], which should produce a tube with all fibres oriented along the tube length. Using micro-CT, Baran et al. [35] found that the orientation of fibres in a pultruded glass fibre reinforced polymer piece had a standard deviation in the two principal axes of 2.53° and 2.56°. However, it must be noted that glass fibres have a different diameter and mechanical properties to carbon fibres, and the piece had a different geometry, so this result may not be comparable. Micro CT was used to confirm that the fibres in our sample appeared unidirectional (S1 Fig) and had a roughly uniform fibre density (S2 Fig), so should produce a uniform dark field.

The diffuser, made from six stacked sheets of P180 silicon carbide (SiC) sandpaper with an average grain size of 82 μm, was placed 2.00 m upstream of the sample stage, which was located *d* = 6.65 m upstream of the detector. The experimental hutch is located approximately 145 m from the source. A photon energy of 35 keV was selected with the double-crystal monochromator. At this energy, the beam dimensions at the sample position are approximately 43 mm x 12mm, resulting in a field-of-view of 1960 x 560 pixels on the detector. The pixel size in the sample plane was 21.5 μm. A pco.edge 5.5 s-CMOS detector (pixel size of 6.5 μm) was used with a Hasselblad Optical System (0.3x objective) and 80 μm Gadox scintillator.

Images of the sample were taken for 25 diffuser positions. The exposure time was 60 ms. A single exposure was used for the sample and diffuser, and 50 images were averaged to create the reference images. The diffuser was moved in a spiral pattern to simulate a pseudo-random pattern that never had identical X or Y components, with steps between positions larger than the size of a speckle. The total experiment time was 23 minutes. The vast majority of the experiment time was spent moving the diffuser motors, which were high precision motors not optimised to move the distances required.

**Fig 3:**
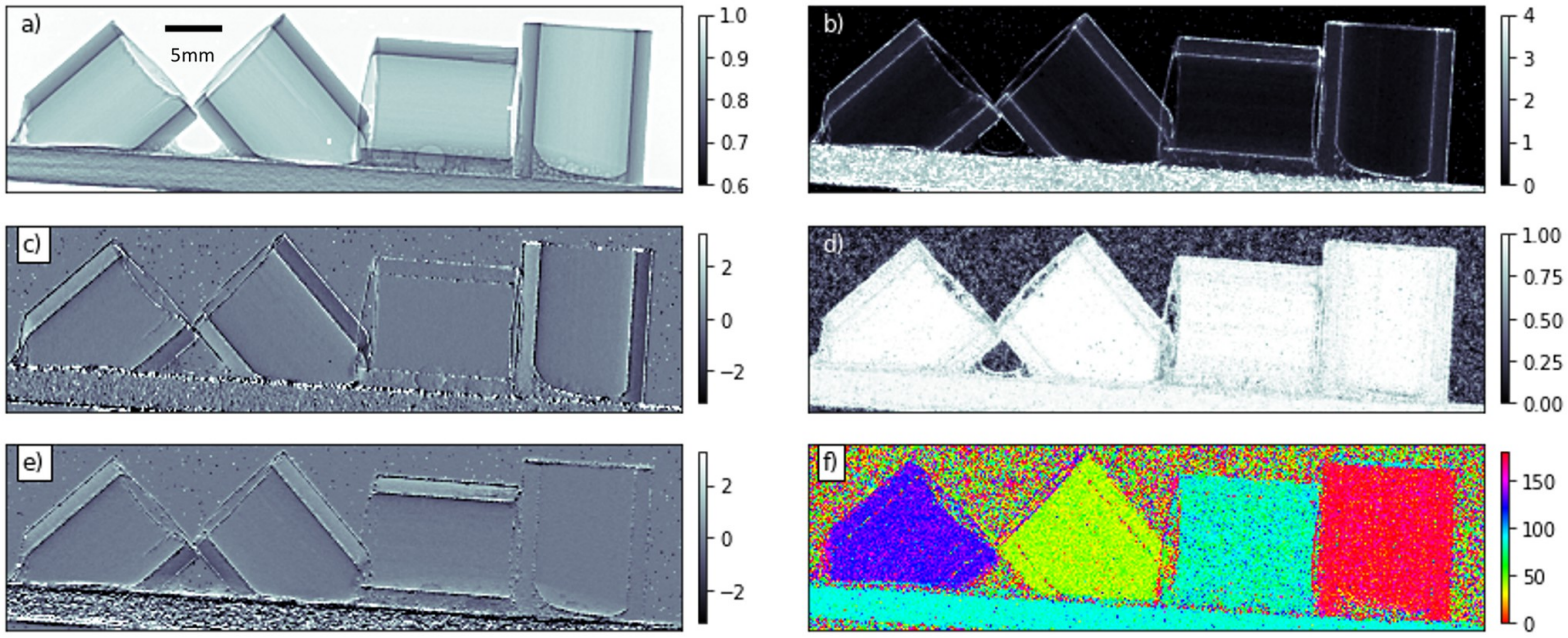
Results of the UMPA-DDF analysis for 25 diffuser positions and a 7 × 7 pixel window. a) Transmittance *T*. c) and e) Horizontal and vertical refraction angles **u**/*d* = (*u*_*x*_/*d*, *u*_*y*_/*d*) in *μ*rad. The right column shows the magnitude, directionality, and orientation of the extracted directional dark-field signal: b) Average kernel width *σ*, in pixels. d) Directionality, given by the eccentricity *ε* of the kernel’s exponential argument. f) Orientation *θ* of the larger scattering axis relative to the horizontal, given in degrees.

The result of our new UMPA-DDF analysis is shown in Fig 3. The three images on the left are the sample transmittance and differential phase-contrast along the horizontal and vertical directions, which can also be extracted with conventional UMPA. The right column of Fig 3 shows the three new signals extracted from the dataset, derived from the parameters above as

$$\begin{array}{cc} \sigma^{2} & =\frac{1}{2}(\sigma_{M}^{2}+\sigma_{m}^{2})=\frac{1}{2}\frac{\alpha_{1}+\alpha_{3}}{\alpha_{1}\alpha_{3}-\alpha_{2}^{2}}, \end{array}$$ (6)

$$\begin{array}{cc} \varepsilon^{2} & =\frac{\sigma_{M}^{2}-\sigma_{m}^{2}}{\sigma_{M}^{2}+\sigma_{m}^{2}}=\sqrt{\frac{{\left(\alpha_{3}-\alpha_{1}\right)}^{2}+4\alpha_{2}^{2}}{{\left(\alpha_{1}+\alpha_{3}\right)}^{2}}}, \end{array}$$ (7)

$$\begin{array}{cc} \theta & =\frac{1}{2}\text{arctan}(\frac{2\alpha_{2}}{\alpha_{3}-\alpha_{1}}). \end{array}$$ (8)

The magnitude of the directional dark-field signal, *σ*, displayed in Fig 3b) shows that the strongest scattering comes from the sample edges and from the wooden sample holder, which is known to produce very strong scattering [20]. The directional aspects of the scattering are displayed in the two lower panels. Fig 3d) is a quantification of the directionality, *ε*. Here the more uniform fibres in the pultruded CFRP tubes can be seen to produce a more directional signal than the wooden holder. Finally, Fig 3f) shows the orientation of the scattering, with the four tubes all scattering orthogonally to the direction of the fibres. The wooden sample holder also scatters orthogonally to the grain direction, which lies along its long axis.

**Fig 4:**
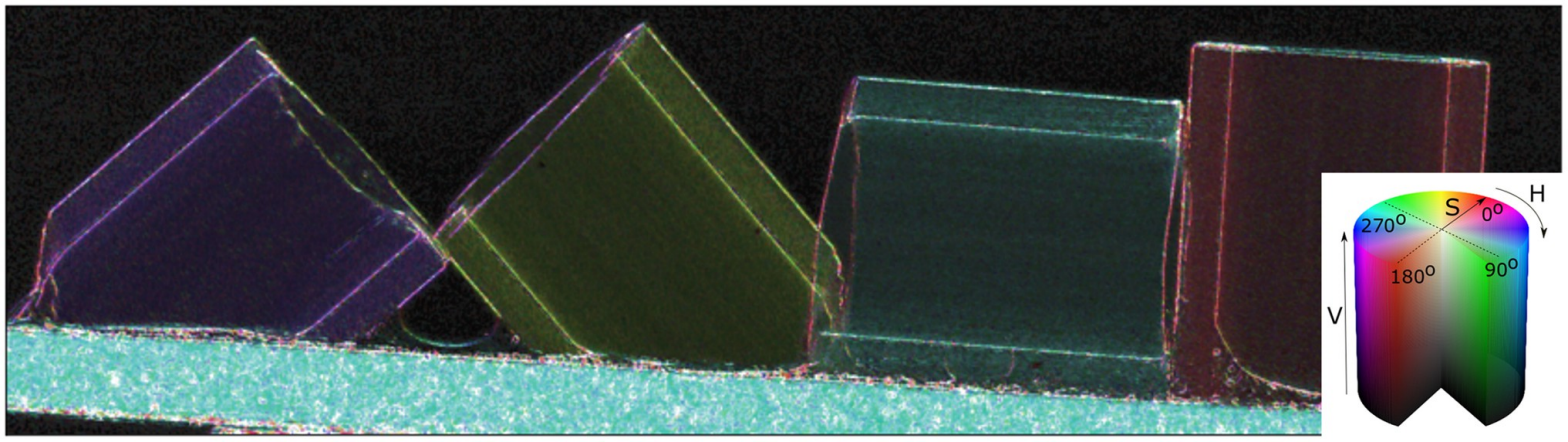
Composite image using the HSV colour space (mapping illustrated with the inset colour-cylinder). The hue *H* shows the orientation *θ* of the scattering, saturation *S* represents the directionality *ε*, and the value *V* shows the mean kernel width *σ*.

Despite containing a significant amount of useful information, the image obtained from the scattering orientation alone can be misleading. For instance, areas of background (and samples with no dark-field) still have an angle associated with them, as do samples which scatter uniformly. To avoid this, we combine the mean kernel width, directionality, and direction information using the HSV (hue/saturation/value) colour space, resulting in Fig 4. The hue represents the orientation of scattering, the saturation the directionality, and the value the mean kernel width. Thus, for instance, features that scatter strongly appear bright, and scattering features with a strong directional component are more colourful than those without a preferred direction, which appear grey.

**Fig 5:**
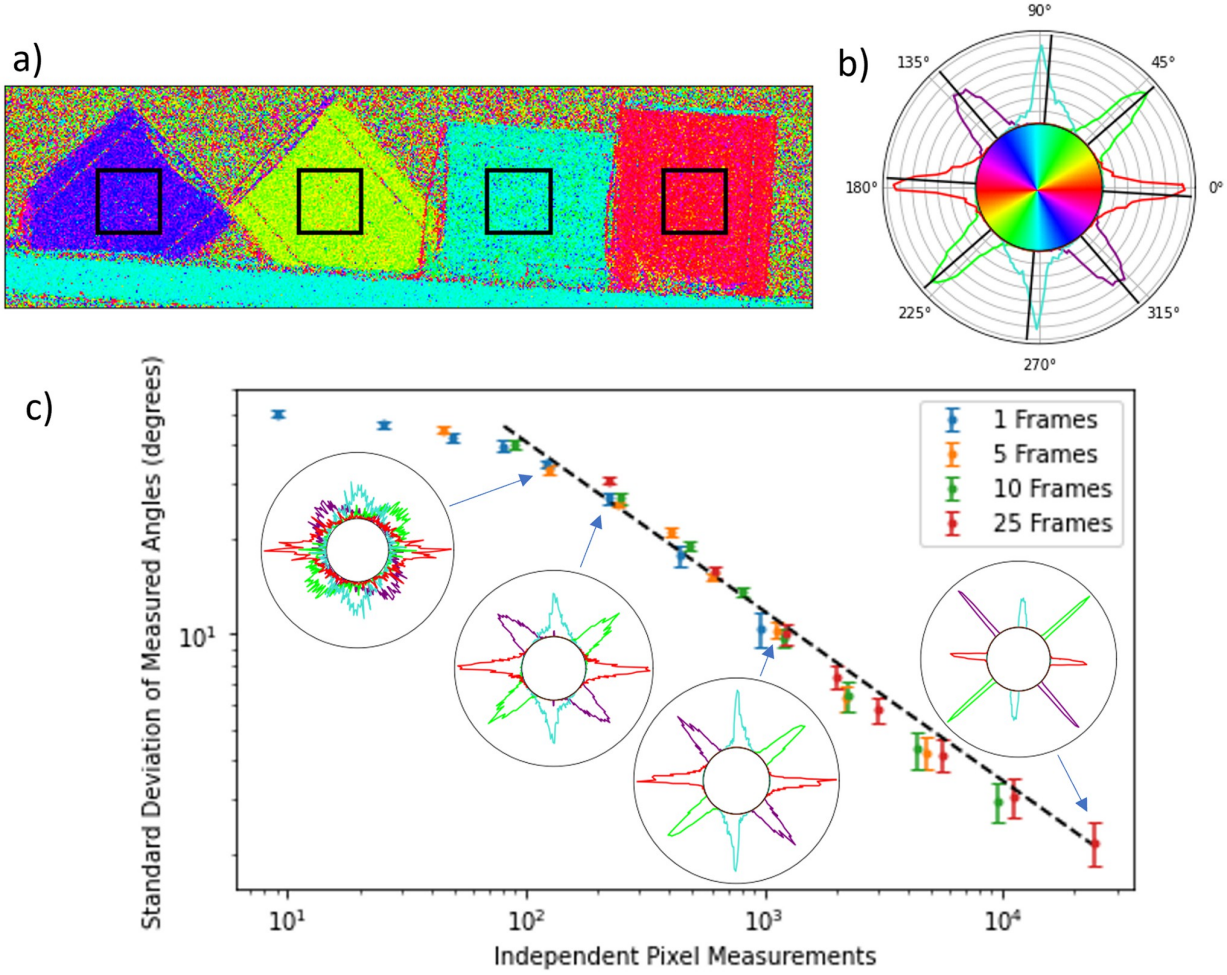
Precision of the retrieved scattering orientation. a) The orientation map highlighting the 150 × 150 pixel regions of interest used for the analysis. b) One example of a polar histogram obtained with 25 diffuser positions and a 7 x 7 pixel analysis window. The central region acts as a colourbar for a). c) Standard deviation of the scattering angles as a function of the number of independent pixel measurements *P*, given by the number of pixels in the window multiplied by the number of diffuser positions used. The error bars are given by the standard error across the four regions. Sample histograms are shown as insets for values of *P* = 125, 225, 961 and 24025. Power-law regression to the data with *P* > 50 yields an exponent of −0.538 ± 0.003 (slope of the black line).

The recovered scattering angles are particularly important for industrial applications. The accuracy and precision with which they are extracted can be evaluated statistically from our images. Fig 5a) indicates 150 x 150 pixel regions of interest selected on the image to quantitatively analyse the distribution of orientations. The resulting polar histograms are displayed in Fig 5b), where the black lines are the expected scattering angle based on the assumption that the scattering occurs perfectly orthogonal to the tubes’ orientation. The tube orientations were measured by taking the gradient between two points at either end of each tube in the transmission image.

It was shown in the past that the precision of the differential phase-contrast signal extracted using the UMPA algorithm depends on the number of independent frames (with different diffuser positions) included in the reconstruction and on the dimensions of the analysis window [30]. Fig 5c) shows that the same behaviour is observed for the extracted scattering angles. The average standard deviation of the four highlighted regions is seen to collapse on a single curve when plotted against the total number of independent measurements, defined as the number of pixels in the reconstruction window multiplied by the number of diffuser positions. The dotted line on the plot is the result of a fit of *σ*_*θ*_ to the curve:

$$\begin{array}{c} \sigma_{\theta }=A\times P^{B}, \end{array}$$ (9)

where *P* is the number of independent pixel measurements. Points with *P* < 50 are excluded from this fit, as the standard deviation values become meaningless due to the histograms wrapping around the full circle. The exponent *B* obtained from this fit is −0.538 ± 0.003, with a reduced $\chi_{\nu }^{2}$ value of 5.1. Given the simple form of the fitted model, which does not include other sources of systematic errors, our result is consistent with the expected $1/\sqrt{P}$ statistics seen in other imaging systems, and in particular for the noise characteristics of the differential phase images of the original UMPA implementation [30]. As discussed previously, Baran et al. [35] found individual fibres can deviate slightly from the expected fibre orientation within pultruded composite materials, which may be the cause of the deviation.

It should be noted that increasing the analysis window size reduces the spatial resolution of the reconstructed image, as information from a larger number of pixels in the initial data is used to find the values for the reconstructed data. As a result, the signals from small features might be lost to large-scale trends. A large analysis window may contain regions of the sample with different scattering profiles, and so a valid solution cannot be found, as there is not a single kernel acting on this window. Using a smaller window would allow for the different scattering profiles in each area to be detected. If only a very small number of diffuser positions are used, a window size that is too small would only produce noise, whereas a larger window would give some correct signal. It is for these reason that using a large window does not produce the same image as using a small window and applying some filter to the results. For our test sample, the accuracy and precision of the orientation of the scattering angle can be reduced to within one degree if the window size is increased up to 31 pixels in size, as shown in Table 1. The errors in the angle of the tube were calculated by assuming an error of ±2 pixels in the points used to measure the gradient.

**Table 1:** Measured tube angles *ϕ* and associated main scattering directions *θ* (both relative to the horizontal axis of the detector). Results were obtained with 25 diffuser positions and analysis window size of 31 × 31 pixels.

Tube	Angle of tube ϕ	Mean scattering angle 〈*θ*〉	Standard deviation of scattering angle	〈*θ*〉 − *ϕ* − 90°
1	(40.7±0.3)°	131.0°	1.5°	0.3°
2	(-48.8±0.3)°	41.3°	1.4°	0.1°
3	(-4.7±0.2)°	84.6°	3.0°	-0.8°
4	(86.5±0.2)°	-3.7°	2.5°	0.2°

The experimental procedure required for UMPA-DDF is in all aspects identical to the one applied with previous applications of UMPA. The new model presented in this paper is able to extract a signal that has been hitherto ignored. It follows that speckle-based directional dark-field imaging shares the known benefits and features of SBI and UMPA. In particular, there are no fundamental obstacles to the translation of the method to laboratory sources, nor to its extension to tomography. The simplicity of the experimental setup suggests that even existing industrial or medical CT systems could be retrofitted to become sensitive to the DDF signal. As we have shown, the precision of the scattering orientation can be increased with many complementary measurements or at the expense of spatial resolution, a trade-off likely to be acceptable in multiple situations.

## Conclusion

SBI is one of multiple emerging X-ray imaging techniques, along with grating-based imaging, ptychography, and edge-illumination techniques, that exploit the redundancy of complementary measurements to improve image quality and add new imaging modalities. We have shown here that a refinement of the scattering model for an experimentally undemanding imaging method has given access to directional scattering information, an imaging modality which will certainly find applications in industry and medicine. Questions that remain thus far unanswered include the type of diffuser or imaging protocol that maximises the robustness and accuracy, as well as the type and number of additional parameters that could be extracted from such datasets. As an example, future models may include the superposition of scattering caused by overlapping features with different orientation, which would be a necessity for translating the presented approach to tomographic imaging, i.e., methods like X-ray tensor tomography.

## Supporting information

S1 File Directional dark field code. Code available at: https://github.com/optimato/UMPA_directional_dark_field DOI:10.5281/zenodo.6981376.
(TXT)

S2 File Updated UMPA Code. Code available at: https://github.com/optimato/UMPA DOI:10.5281/zenodo.6984740.
(TXT)

S3 File Experimental data. A copy of the data taken at ID19 Beamline of ESRF Synchrotron is available at DOI:10.15151/ESRF-DC-745664887.
(TXT)

S1 Fig Radial tomogram slice. A small section from the same CFRP tube used to make the sample was scanned in a Zeiss-Xradia Versa 510 CT system. The 4x objective was used with an unbinned detector, and the geometric magnification set to give a pixel size of 1.03 μm. The accelerating voltage was 80 kV and the power was 7 W. 4501 projections with 10 s of exposure were taken. A radial slice was taken with the internal edge of the tube shown in the top part of the figure. The fibres can be seen to run parallel to this edge. The bright spots in the image are tiny pieces of dense material (most likely metal shavings) which have been accidentally introduced during the manufacturing process but should not effect the dark field signal. Moire fringes are visible showing there are some slight deviations in the fibre orientation orthogonal to the slice.
(TIF)

S2 Fig Transverse tomogram slice. A radial slice taken from the tomogram described above, showing that beside a few small regions, the overall fibre density within the sample is fairly uniform. There is a slightly lower density of fibres in the region near the edge, and in some small regions in the center of the field of view where a contaminant particle can be seen.
(TIF)
