# Supplementary figures and images for: X-ray directional dark-field imaging using Unified Modulated Pattern Analysis
Source: PLoS One. 2022 Aug 29;17(8):e0273315. doi: 10.1371/journal.pone.0273315 (PMC9423625; doi:10.1371/journal.pone.0273315)

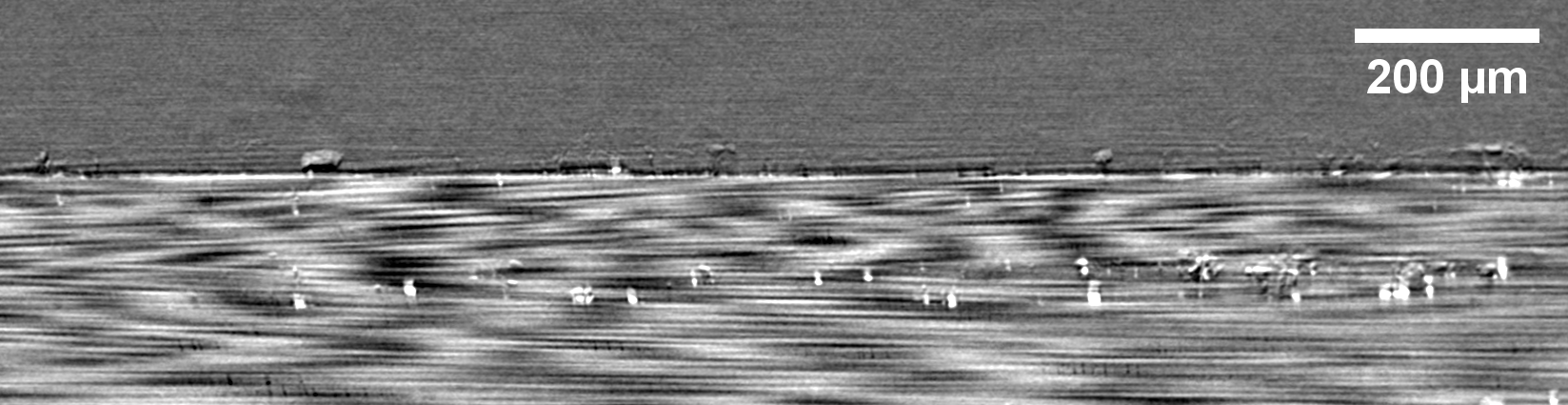

Supplement: S1 Fig — A small section from the same CFRP tube used to make the sample was scanned in a Zeiss-Xradia Versa 510 CT system. The 4x objective was used with an unbinned detector, and the geometric magnification set to give a pixel size of 1.03 μm. The accelerating voltage was 80 kV and the power was 7 W. 4501 projections with 10 s of exposure were taken. A radial slice was taken with the internal edge of the tube shown in the top part of the figure. The fibres can be seen to run parallel to this edge. The bright spots in the image are tiny pieces of dense material (most likely metal shavings) which have been accidentally introduced during the manufacturing process but should not effect the dark field signal. Moire fringes are visible showing there are some slight deviations in the fibre orientation orthogonal to the slice. (TIF) [file pone.0273315.s004.tif]

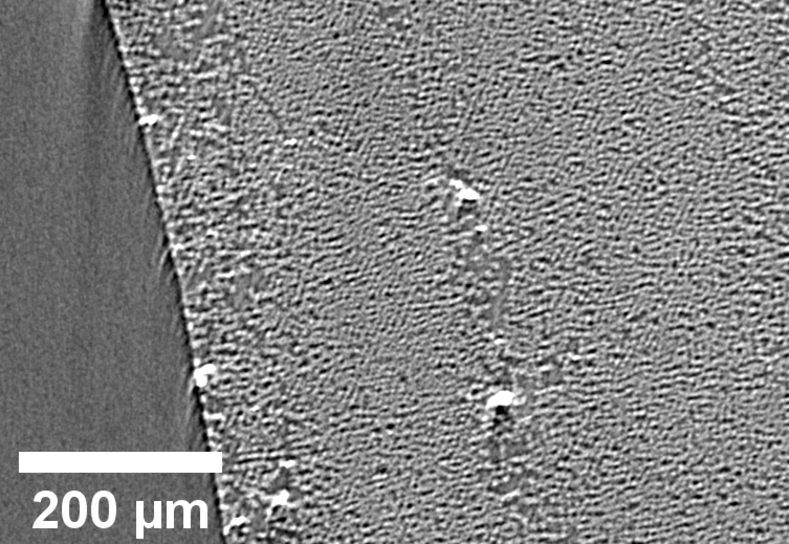

Supplement: S2 Fig — A radial slice taken from the tomogram described above, showing that beside a few small regions, the overall fibre density within the sample is fairly uniform. There is a slightly lower density of fibres in the region near the edge, and in some small regions in the center of the field of view where a contaminant particle can be seen. (TIF) [file pone.0273315.s005.tif]
